# Near-anoxia induces immobilization and sustains viability of sperm stored in ant queens

**DOI:** 10.1038/s41598-023-29705-7

**Published:** 2023-03-01

**Authors:** Ayako Gotoh, Mika Takeshima, Ken-ichi Mizutani

**Affiliations:** 1 Department of Biology, Faculty of Science and Engineering, Konan University, Kobe, 658-8501 Japan; 2 Institute for Integrative Neurobiology, Konan University, Kobe, 658-8501 Japan; 3 Suntory Rising Stars Encouragement Program in Life Sciences (SunRiSE), Kobe, Japan; 4 Laboratory of Stem Cell Biology, Graduate School of Pharmaceutical Sciences, Kobe Gakuin University, Kobe, 650-8586 Japan

**Keywords:** Reproductive biology, Animal physiology

## Abstract

After copulation, insect females store sperm in a spermatheca for some duration until fertilization. At the beginning of their adult lives, ant queens can preserve numerous viable sperm cells from copulation for over ten years. However, the key factors influencing long-term sperm storage have not been identified. Here we show that the spermathecal environment is nearly anoxic, which induces sperm immobilization. Furthermore, mitochondrial respiratory inhibitors suppress sperm motility, suggesting that sperm immobilization may be caused by a shortage of ATP generated from only glycolysis under near-anoxic conditions. Sperm immobilization is not induced by acidification via glycolytic metabolism because the spermathecal fluid is not acidic. Finally, we show that artificial anoxic conditions rather than aerobic conditions sustain viable sperm cells. Therefore, near-anoxia is a key factor influencing long-term sperm storage in ant queens. The viability of sperm cells under artificial anoxia, however, is lower than that of those dissected immediately from queens. Moreover, the immotile sperm cells under more than 4 h of anoxia do not begin swimming after aerobic exposure, unlike those under anoxic conditions for less than 2 h. This finding indicates that factors other than anoxia are also necessary for long-term sperm preservation.

## Introduction

Various unique reproductive strategies for reproductive success are known in both sexes of sexual organisms. Among them, many species of various taxa have evolved the strategy of sperm storage between copulation and fertilization inside the female reproductive tract^1^. The duration of sperm storage ranges from temporary to over a decade among various species. Ant queens copulate only at the beginning of their adult lives and use the sperm for fertilization until they die; therefore, they can maintain viable sperm cells for the same duration as their life span. Because queens can live for more than 10 years in many ant species^2^, their capacity for sperm maintenance is pronounced. However, the physiological mechanisms by which ant queens maintain viable sperm cells are still unclear.

Ant queens maintain sperm cells inside their sperm storage-sac (spermathecal reservoir)^3^. It consists of mitochondria-rich columnar epithelia and flattened epithelia with few organelles^3,4^. The flattened epithelium is considered to not affect the sperm storage function because of its ultrastructure. In contrast, the columnar epithelium is considered to perform a transporting function between inside and outside of the spermathecal reservoir. It has been reported that sperm cells stored in the spermathecal reservoir are immobilized^5^. Furthermore, immobilization has also been observed during sperm transfer from the bursa copulatrix to the spermathecal reservoir via the spermathecal duct soon after copulation^5^. Sperm immobilization is considered to have significant advantages for long-term sperm storage in avoiding the risk of sperm damage and inhibiting the production of reactive oxygen species if they use oxygen to produce adenosine triphosphate (ATP). Therefore, understanding the mechanism of sperm immobilization could elucidate the mechanism of prolonged sperm storage in ant queens.

Many studies have reported on factors that induce sperm motility after male ejaculation in many animals, e.g., serine proteases in silkworms^6^, and change in extracellular osmolality inducing a change in intracellular ion concentration in many teleost species^7^. However, studies on the mechanisms that regulate the motility of sperm stored in females are limited^8^.

In this study, we show that near anoxia in the spermathecal reservoir is a key factor influencing sperm immobilization, and artificial anoxia rather than aerobic conditions can sustain viable sperm cells.

## Results

### Oxygen concentrations in the spermathecal reservoir and other tissues

**Figure 1:**
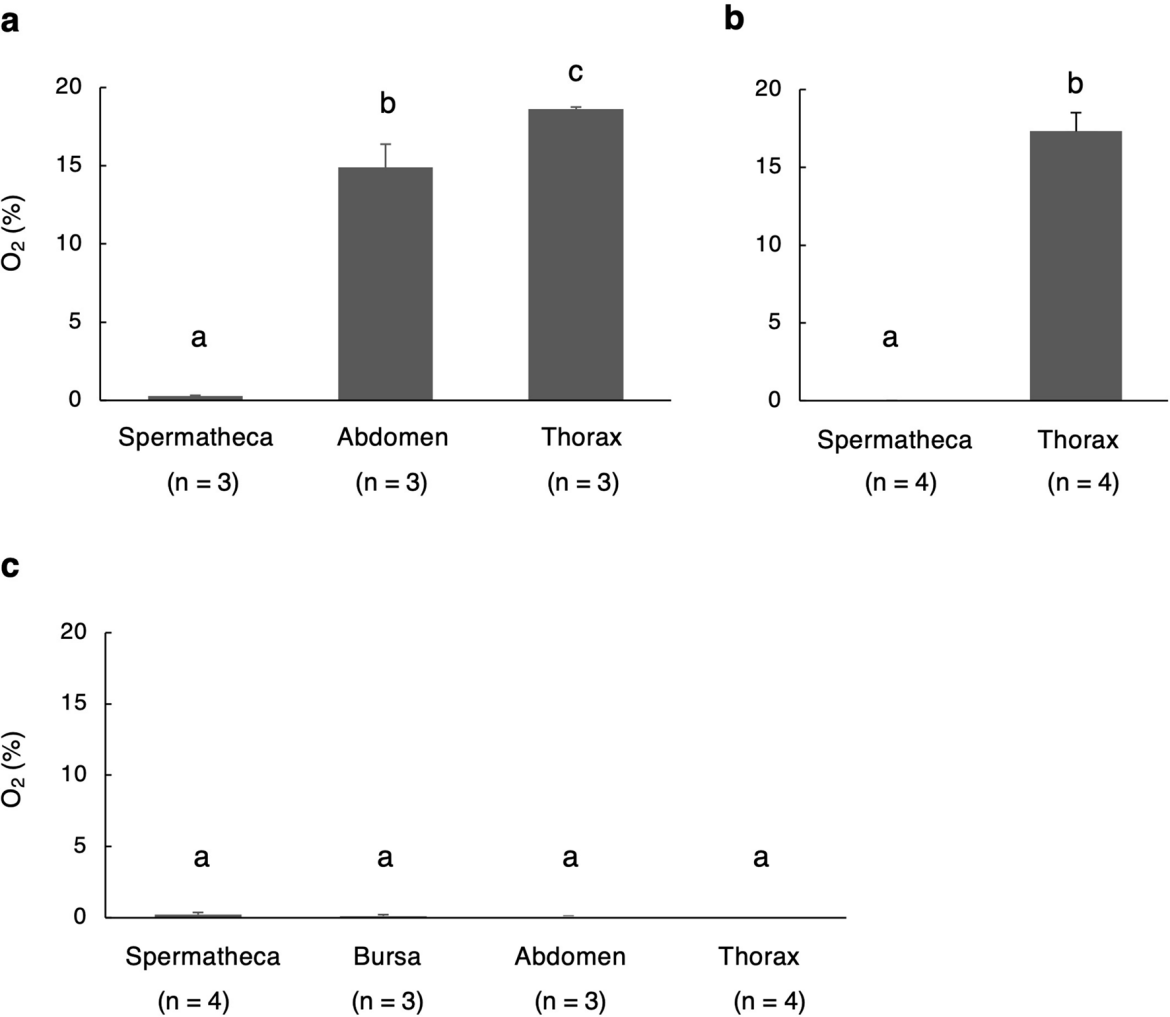
Tissue oxygen concentration in (**a**) *Crematogaster osakensis* queens at 1 year, (**b**) *Lasius hayashi* queens at 7 months after the beginning of sperm storage (or nuptial flight), and (**c**)* C*. *osakensis* queens soon after nuptial flight. Error bars represent standard deviation. Different alphabetical symbols indicate statistical significance at *p* < 0.05 based on Tukey’s post-hoc test in (**a**) and (**c**) and Mann–Whitney *U* test in (**b**).

Oxygen concentrations in the spermathecal reservoir of *Crematogaster osakensis* and *Lasius hayashi* mated queens were 0.284 ± 0.036% (mean ± SD, n = 3) and zero (n = 4), respectively (Fig. 1a and b). These concentrations were extremely low compared with those in the abdomen (mean ± SD, 14.914 ± 1.470%, for *C. osakensis*, n = 3) and thorax (mean ± SD, 18.611 ± 0.142%, for *C. osakensis*, n = 3; and 17.330 ± 1.182%, for *L. hayashi,* n = 4) (Tukey’s post-hoc test, for *C. osakensis*: spermatheca–abdomen, *p* < 1e−5; spermatheca–thorax, *p* < 1e−5; and abdomen–thorax, *p* = 0.011; Mann–Whitney *U* test, for *L. hayashi*: spermatheca–thorax, *p* = 0.021). Additionally, in *C. osakensis* queens collected soon after mating (on the night of the nuptial flight), oxygen concentrations both in the spermathecal reservoir (mean ± SD, 0.211 ± 0.189%, n = 4) and other tissues, the bursa copulatrix (mean ± SD, 0.125 ± 0.114%, n = 3), abdomen (mean ± SD, 0.048 ± 0.083%, n = 3), and thorax (mean ± SD, 0.003 ± 0.006%, n = 4), were extremely low (Tukey’s post-hoc test: spermatheca–bursa, *p* = 0.792; spermatheca–abdomen, *p* = 0.345; spermatheca–thorax, *p* = 0.134; bursa–abdomen, *p* = 0.861; bursa–thorax, *p* = 0.571; and abdomen–thorax, *p* = 0.960; Fig. 1c).

### Artificial anoxic conditions induce sperm immobilization

In subsequent analyses, we used mated *C. osakensis* queens. This species is highly useful for studying sperm-storage mechanisms, in that several thousand queens can be collected during the nuptial flight, and can be kept easily. *C. osakensis* sperm cells extracted from the spermathecal reservoir are known to begin swimming in phosphate-buffered saline (PBS)^5^. To investigate the effect of near-anoxia on sperm immobilization, we created hypoxic PBS using an O_2_-absorbing agent, AnaeroPack® Kenki pouch and anoxic PBS using an O_2_ scavenger (39.44 mM sodium sulfite).

Sperm cells began swimming immediately after exposure to the aerobic PBS control (n = 5). In contrast, they were immotile soon after exposure to the hypoxic PBS (0.4–0.5%), and then gradually began swimming 2–4 min later, which may be caused by atmospheric oxygen dissolution in the artificial hypoxic PBS with time (n = 4, Supplementary Movie 1).

**Figure 2:**
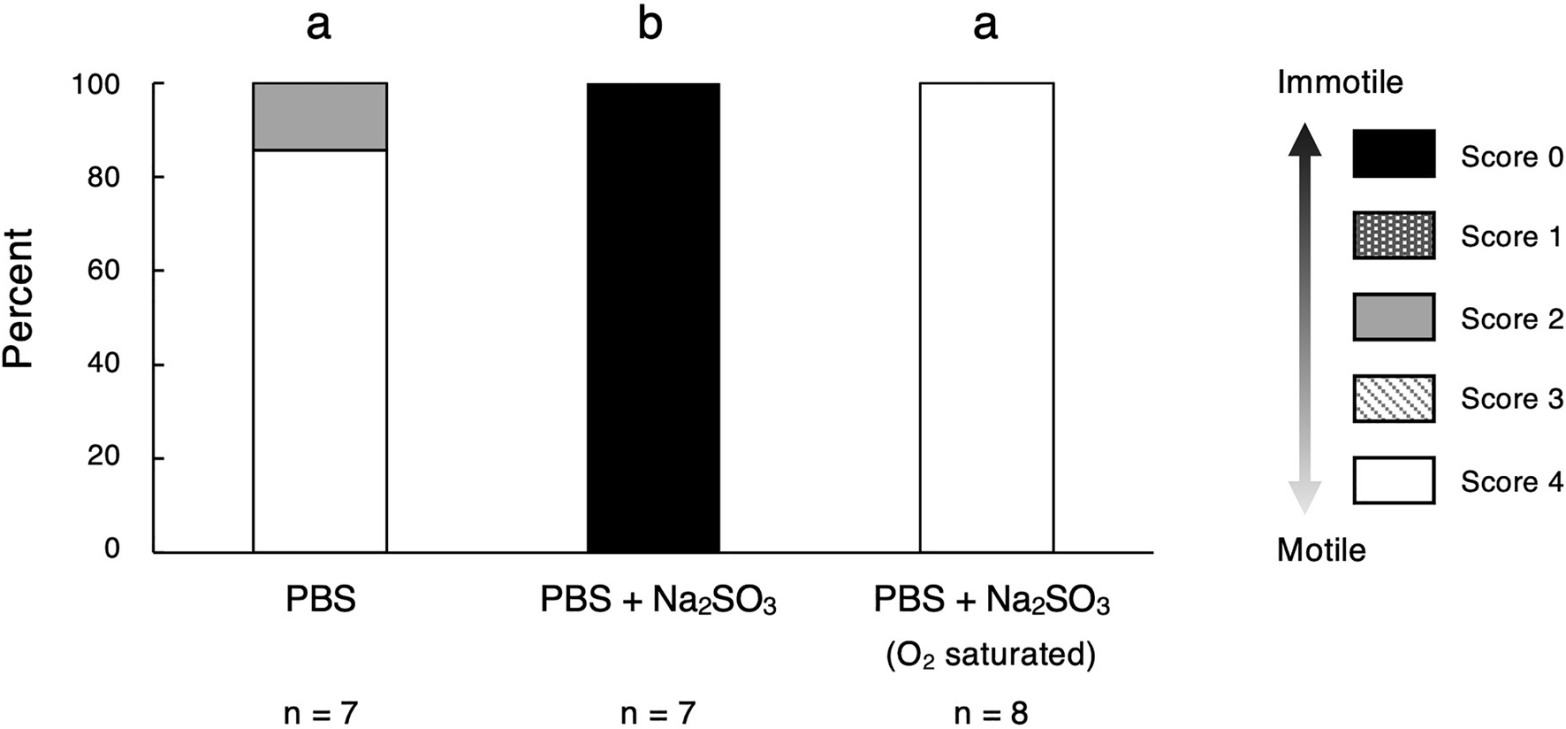
Sperm motility in the aerobic PBS control (21% O_2_), the anoxic PBS condition (0% O_2_) created by adding sodium sulfite and an oxygen-saturated sodium sulfite solution in PBS (21% O_2_). Different alphabetical symbols indicate statistical significance at *p* < 0.05, based on Tukey’s post-hoc test.

Unlike in the aerobic PBS control, complete sperm immobilization was observed when sodium sulfite was used to create anoxic conditions (Tukey's post-hoc test: PBS–PBS containing sodium sulfite, *p* < 1e−7; Fig. 2 and Supplementary Movie 2). To reject the possibly that sperm death caused this immobility, we added double the quantity of aerobic PBS buffer to the immotile sperm cells: all of the sperm cells began swimming, indicating that they were not dead but only immobilized under the anoxic conditions. Furthermore, sperm cells exposed to an oxygen-saturated PBS solution containing sodium sulfite (oxygen concentration of 21%, matching that of air), which was obtained by allowing the solution to stand for one day were as motile as those in the aerobic PBS solution (Tukey's post-hoc test: PBS–oxygen-saturated PBS containing sodium sulfite, *p* = 0.413; oxygen-saturated PBS containing sodium sulfite–PBS containing sodium sulfite, *p* < 1e−7; Fig. 2). This indicated that adding sodium sulfite to PBS does not affect sperm immobilization.

### Effect of glycolytic and mitochondrial respiratory pathway inhibitors on sperm motility

**Figure 3:**
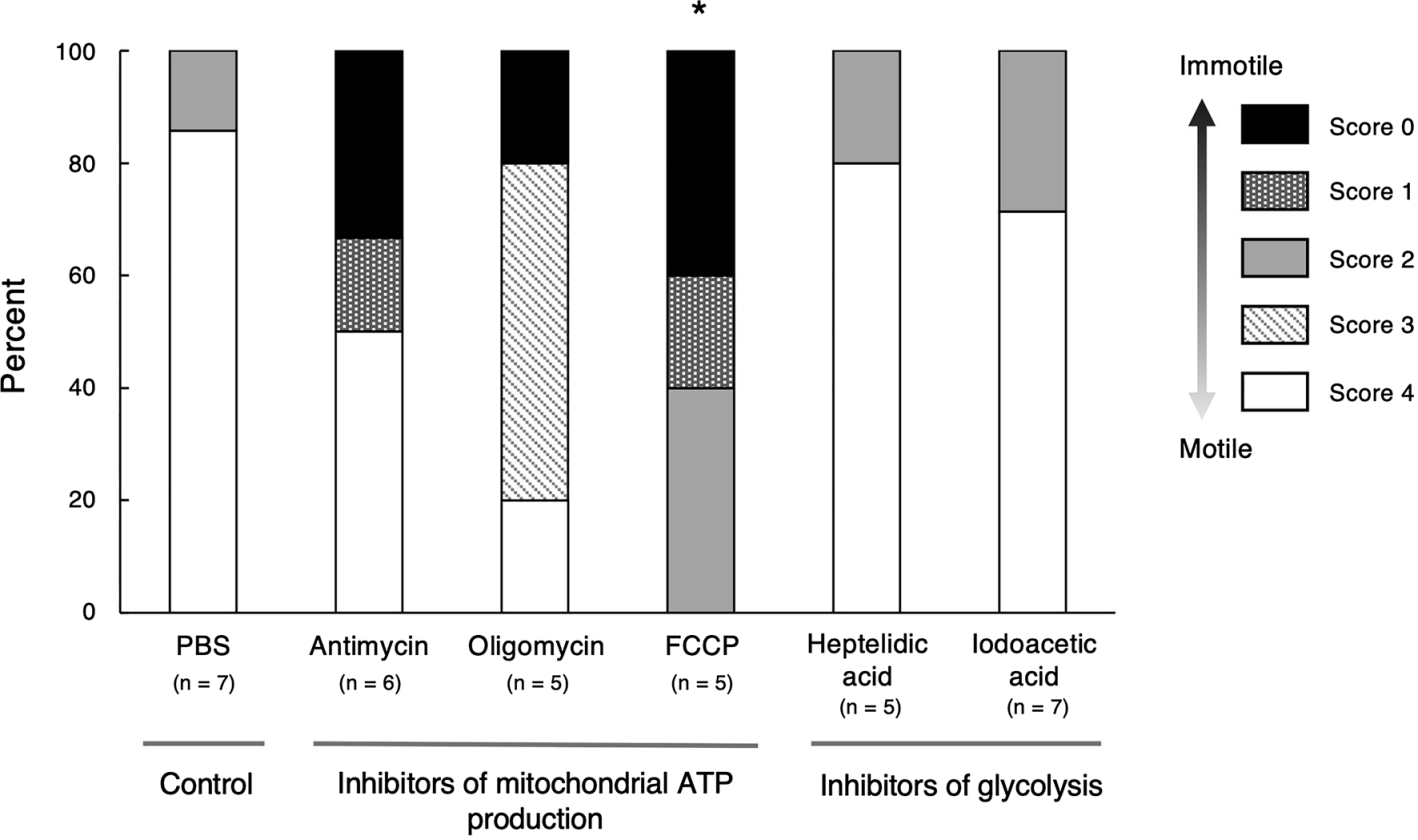
Effect of mitochondrial ATP production and glycolysis inhibitors on sperm motility. The asterisk indicates a statistically significant difference compared with the control at *p* < 0.05, based on a Kruskal–Wallis *H* test and Steel’s multiple comparison Wilcoxon tests.

We hypothesized that sperm immobilization under hypoxic and anoxic conditions was induced by a shortage of ATP originating from only glycolysis. Therefore, we investigated the effect of glycolytic and mitochondrial respiratory pathway inhibitors on sperm motility using sperm cells extracted from *C*. *osakensis* queens. Sperm motility was suppressed by mitochondrial respiratory inhibitors—antimycin, oligomycin, and carbonyl cyanide 4-(trifluoromethoxy) phenylhydrazone (FCCP)—although only FCCP had a statistically significant effect (Kruskal–Wallis *H* test and Steel’s multiple comparison Wilcoxon tests comparing control PBS and inhibitors: antimycin, *t* = 1.57, *p* = 0.390; oligomycin, *t* = 1.92, *p* = 0.208; and FCCP, *t* = 2.89, *p* = 0.018; Fig. 3). The glycolytic pathway inhibitors heptelidic acid and iodoacetic acid did not affect sperm motility (Kruskal–Wallis *H* test and Steel’s multiple comparison Wilcoxon tests comparing control PBS and inhibitors: heptelidic acid, *t* = 0.25, *p* = 0.999, and iodoacetic acid, *t* = 0.628, *p* = 0.962; Fig. 3).

### Sperm cellular energy status under anoxic and aerobic conditions

**Figure 4:**
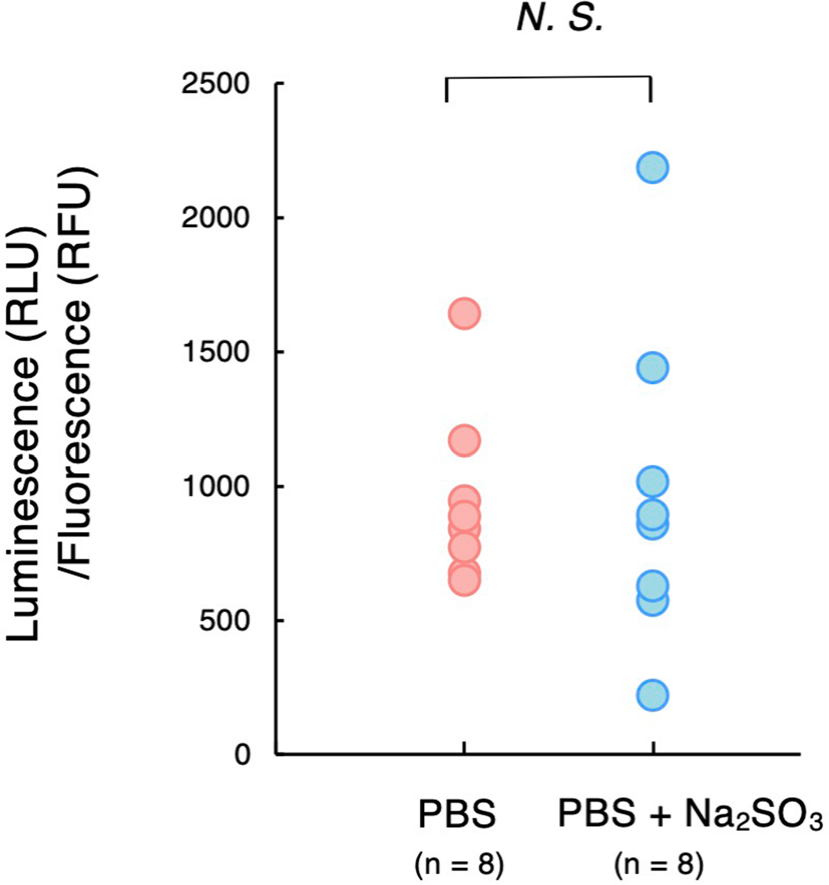
Sperm ATP levels calibrated by the number of cells in the aerobic PBS control and anoxic PBS (created by adding sodium sulfite). RLU, relative luminescence unit; RFU, relative fluorescence unit.

**Figure 5:**
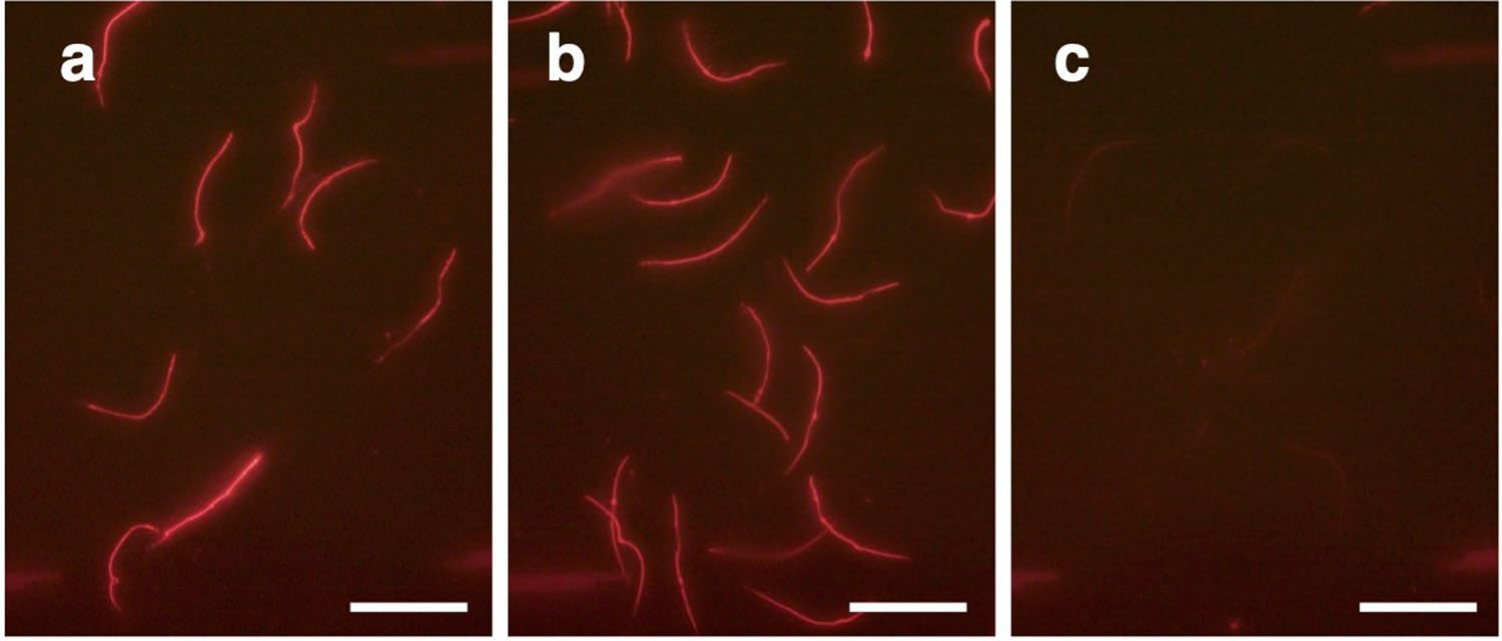
Mitochondrial membrane potential of sperm in (**a**) aerobic PBS control, (**b**) anoxic PBS (after adding sodium sulfite), and (**c**) aerobic PBS containing buffer FCCP as negative control. Scale bars indicate 50 µm.

We assessed ATP levels and mitochondrial activity in sperm cells extracted from *C*. *osakensis* queens under anoxic and aerobic conditions. Motile sperm cells induced by PBS did not produce more ATP than immobilized sperm cells exposed to PBS containing sodium sulfite solution (mean ± SD, 943.4 ± 304.1 relative luminescence/fluorescence units in PBS solution, and 975.4 ± 565.0 relative luminescence/fluorescence units in PBS containing sodium sulfite solution; Mann–Whitney *U* test, *W* = 34, *p* = 0.8785; Fig. 4), and membrane potentials of sperm mitochondria were similar under anoxic and aerobic conditions (Fig. 5).

### The pH of spermathecal fluid and its effect on sperm motility

**Figure 6:**
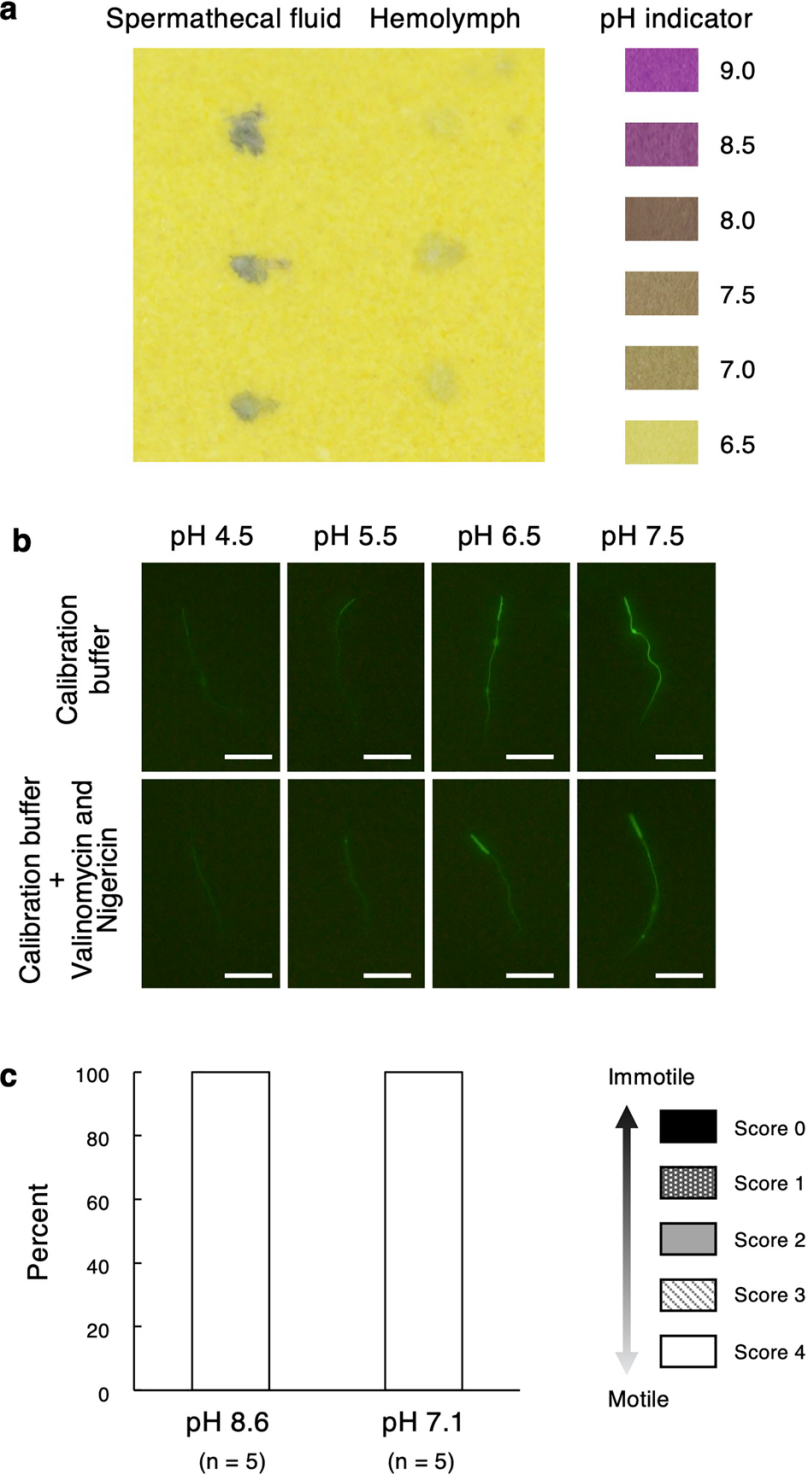
(**a**) pH of the spermathecal fluid and hemolymph. (**b**) Effect of extracellular fluid pH on intracellular pH of sperm. Fluorescence intensity of BCECF-loaded sperm exposed to various pH-calibrated buffers and those containing valinomycin and nigericin (to equilibrate the pH inside and outside cells). Scale bars indicate 20 µm. (**c**) Effect of pH mimicking spermathecal fluid (pH 8.6) and hemolymph (pH 7.1) on sperm motility.

In mated queens of *C*. *osakensis*, the pH of spermathecal fluid was determined using pH paper to assess whether it presents an acidic environment under near-anoxic conditions of the spermathecal fluid. The pH in the reservoir was approximately 8.5, which was higher than that of the hemolymph (approximately 7.0; Fig. 6a). The intracellular sperm pH assessed by fluorescent pH indicator increased in proportion to extracellular fluid pH (Fig. 6b). Furthermore, the fluorescence intensity was similar in response to extracellular fluid with and without valinomycin and nigericin (which equalize intra- and extra-cellular pH) (Fig. 6b). Therefore, extracellular pH equalized intracellular sperm pH, and the intracellular pH of the sperm preserved in the spermathecal reservoir fluid was not likely to be acidic.

Sodium chloride solutions adjusted to pH 8.6 and 7.1 mimicking spermathecal reservoir fluid and hemolymph pH, respectively, did not induce sperm immobilization (Mann–Whitney *U* test, *W* = 12.5, *p* = 1; Fig. 6c).

### Effect of preservation under aerobic and anoxic environments on sperm viability and physiology

**Figure 7:**
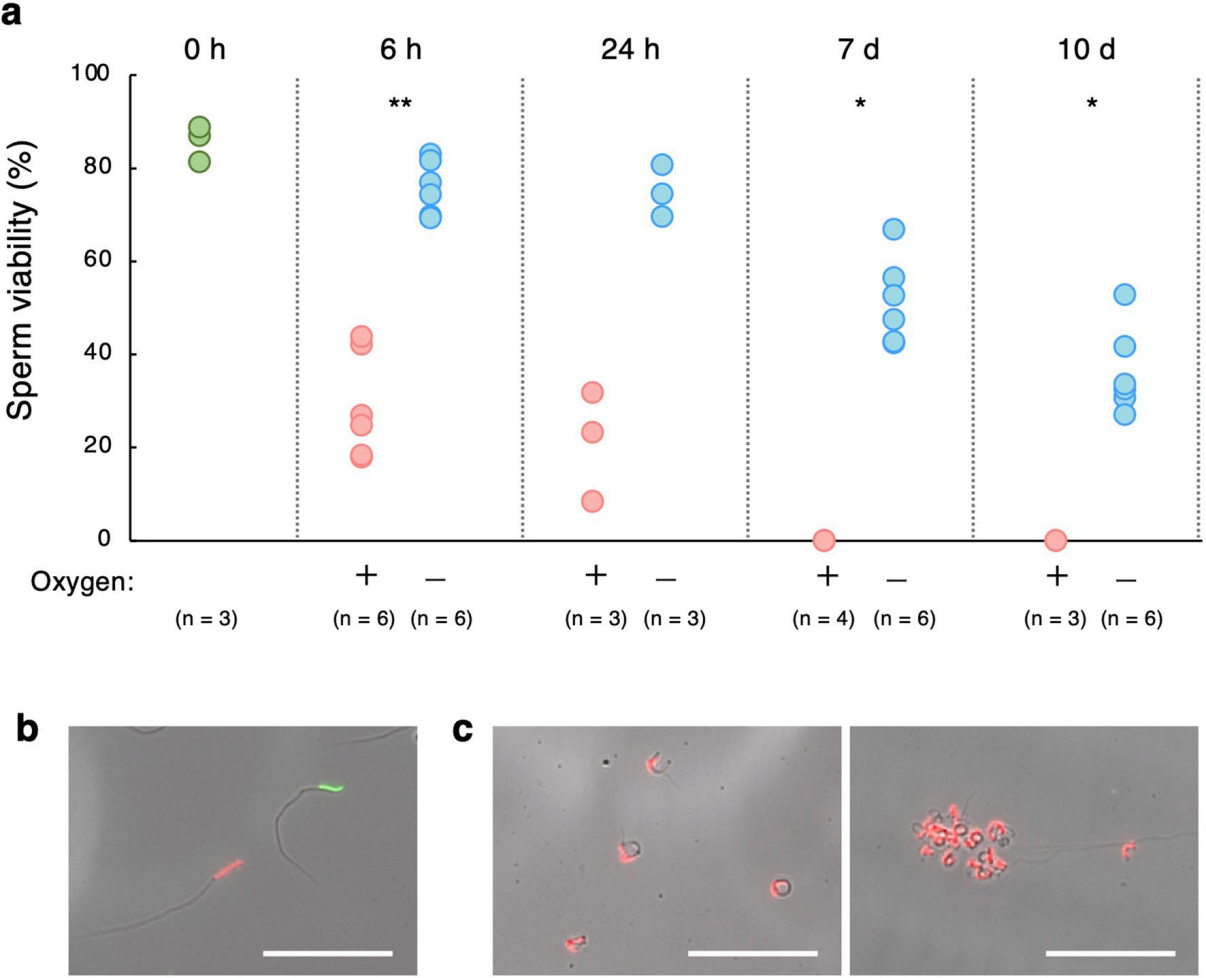
(**a**) Sperm viability under aerobic (+) and anoxic (after adding sodium sulfite) (−) PBS conditions. Asterisks indicate statistical significance based on Mann–Whitney *U* tests (* p < 0.05, **p < 0.005). (**b**) Image of surviving sperm stained green and dead sperm stained red under artificially anoxic conditions for 7 days. (**c**) Images of dead sperm cells with abnormal shape (left), and sperm aggregation (right). Scale bars indicate 50 µm.

The effect of anoxia on sperm viability in *C*. *osakensis* was investigated using a dual fluorescent staining. The viability of sperm preserved for 6 h, 24 h, 7 days, and 10 days under anoxic conditions was higher than that under aerobic conditions, although this was not statistically significant at 24 h (Mann–Whitney *U* test: 6 h, *W* = 0, *p* = 0.002; 24 h, *W* = 0, *p* = 0.100; 7 days, *W* = 0, *p* = 0.011; and 10 days, *W* = 0, *p* = 0.026; Fig. 7a). In fact, under aerobic conditions in both the 7 days and 10 days treatments, sperm aggregation and abnormalities were observed (Fig. 7b, c), and no sperm survived (Fig. 7a).

Sperm cells preserved in artificial anoxic conditions for 1 and 2 h began swimming when aerobic PBS was added. However, we observed no motility in the 8, 18, and 24 h preservations, and very few were motile in the 4 h preservation (n = 3, Supplementary Movie 3).

## Discussion

### Sperm cells are stored in near-anoxic conditions and remain immotile

In this study, we found that the oxygen concentration in the spermathecal reservoir was extremely low in the two ant species, particularly in *L. hayashi* queens. Inserting oxygen probes into the small spermathecal reservoirs of *C. osakensis* queens was technically difficult, and oxygen could diffuse into the spermathecal fluid during the insertion. Therefore, the true oxygen concentration may be lower than our data reflects. This indicates that they have a powerful oxygen removal system inside the spermathecal reservoir. Because the two species belong to different subfamilies, the near-anoxic conditions in the spermathecal reservoir may be a general feature in ants.

We also demonstrated, using two methods, that artificial hypoxic and anoxic environments induced sperm immobilization. Although many studies have examined sperm motility in artificial fluids in insects, it has been challenging to confirm that sperm motility actually occurs in vivo because of the difficulty in measuring the environment around sperm in the small reproductive tract^9^. Therefore, this study is one of the few that link the internal environment of the reproductive organ and the effect of the factor on sperm motility by using ant queens with a large spermatheca and a pronounced sperm storage ability among insect species.

### Mechanism of immobilization during sperm transfer into the spermathecal reservoir

Our previous study revealed that sperm cells are immotile when they are transferred to the spermathecal reservoir of the queen soon after copulation^5^. In this study, we have demonstrated that the spermathecal reservoir, bursa copulatrix, and the non-reproductive tissues, such as the abdomen and thorax, in ant queens were nearly anoxic soon after the nuptial flight. Consequently, sperm immobilization during transfer is presumed to be caused by the near-anoxic conditions throughout the body. Ant queens probably use their flight muscles intensively during the nuptial flight. Thus, the anoxic conditions throughout the body may result from oxygen consumption during that flight. However, there are also ant species in which the queen copulates with males around their natal nest without nuptial flight^10^. Thus, assessing the oxygen concentration and sperm motility soon after copulation in these species is also necessary.

It is unclear whether sperm cells are motile after they are released from the spermathecal reservoir for fertilization^5^. It was technically challenging to measure oxygen concentration inside the spermathecal duct and bursa copulatrix because of their narrow lumens; therefore, we could not assess sperm motility for fertilization in terms of the oxygen level of the immediate environment.

### Mechanism of sperm immobilization under anoxic conditions

It was considered that ATP for sperm motility was produced by oxidative phosphorylation through aerobic respiration in animal species; however, insects such as the insects *Tribolium castaneum*^11^ and *Aleochara bilineata*^12^ and the mammalian mouse *Mus musculus*^13^ have been found to use a glycolytic system. In this study, sperm motility was restricted by respiratory inhibitors, not glycolytic pathway inhibitors. Aerobic respiration through glycolysis, the tricarboxylic acid cycle, and oxidative phosphorylation together yields 38 mol of ATP, whereas glycolysis alone generates only 2 mol of ATP per mol of glucose. Sperm immobilization in the spermathecal reservoir of an ant queen may be caused by a shortage of ATP generated from only glycolysis. However, we did not find more intracellular ATP under aerobic than anoxic conditions. This suggests that ATP production may be balanced by consumption (for sperm motility) under aerobic conditions. A mitochondrial membrane potential was detected in the sperm under anoxic conditions. It has various roles besides ATP production, such as antiviral signaling^14^ and generating a pulling force for translocating newly synthesized proteins into the mitochondrial matrix^15^. In the sperm of ants, elongated mitochondria occupy a large part of the flagellum as well as those of other insects^9,16^, although ant sperm cells are stored in nearly anoxic conditions. Therefore, the presence of a mitochondrial membrane potential in nearly anoxic conditions is necessary for sperm functions, which requires further research.

Usually, either exogenous or endogenous substrates, such as sugars and lipids, are necessary as an energy resource for sperm motility. The ant sperm cells ejected from spermathecae were motile in the PBS buffer, which did not contain sugars and lipids, indicating the presence of an endogenous energy substrates. Also, the glycolytic pathway inhibitors heptelidic acid and iodoacetic acid, which inhibit the catalysis of glyceraldehyde 3-phosphate to 1,3-bisphosphoglyceric acid, did not induce sperm immobilization. This suggests that sperm cells accumulate energy substrates in the pathway after the point of 1,3-bisphosphoglyceric acid in glycolysis and the tricarboxylic acid cycle.

### Sperm immobilization is not related to the pH of the spermathecal fluid

Female Japanese quails can store sperm for 11–12 days in sperm storage tubules after mating, and the stored sperm cells are also immobilized in the hypoxic environment of the sperm storage tubules^8^. However, the sperm immobilization process is different from ants. In Japanese quails, lactic acid is accumulated through the glycolytic pathway in the hypoxic conditions of the sperm storage tubules, which decreases the intracellular sperm pH. Because the dynein ATPase activity of sperm is generally inhibited by acidic pH, the sperm of Japanese quails cannot swim while in the sperm storage tubules^8,9^. However, our study revealed that the pH of the spermathecal fluid was high (approximately 8.5) and that intracellular sperm pH was related to extracellular pH in ants. Therefore, sperm immobilization could not be caused by acidification. This also suggests that the spermathecal reservoir regulates luminal pH and eventually inhibits acidification of stored sperm in a nearly anoxic environment. In ant queens, the columnar epithelial cells of the spermathecal reservoir are considered to have transport functions^3^. In cancer cells, transporters play an important role in inhibiting lactic acid accumulation under hypoxic conditions^17^. Therefore, a pH regulation system mediated by transporters and channels related to transition protons and lactic acid should exist in both the spermathecal epithelium and sperm plasma membrane.

An NaCl solution, which mimicked the spermathecal fluid pH, did not induce sperm immobilization, indicating that a high pH does not affect sperm motility in ants. In this experiment, an NaCl solution, which has simpler contents than PBS, could induce sperm motility, suggesting that ion components only in PBS are also not related to the induction of sperm motility.

### Evolution of a sperm storage system in Hymenoptera

The order Hymenoptera is a large group, and sperm storage duration ranges from several weeks or months in solitary species to several decades in ants that have developed prominent eusociality. Honeybee (*Apis mellifera*) queens can store sperm cells for 2–4 years^18^, which is a long duration, second only to ants—although they belong to a different lineage. Near anoxia, high pH of the spermathecal fluid, and immobility of stored sperm in *A. mellifera* queens have been reported^19–21^. It is suggested that the two lineages evolved a common mechanism for sperm maintenance. In honeybee queens, the spermathecal reservoir is surrounded by a tracheal network^22–24^, which is considered to suppress oxygen spread to the spermathecal reservoir and maintain near anoxia^21^. However, ant spermathecae possess no tracheal network^3^. In both honeybees and ants, abundant mitochondria are present in the columnar epithelium of the spermathecal reservoir^3^; therefore, they may consume oxygen and maintain near anoxia inside the reservoir. To our knowledge, studies investigating sperm physiology and molecular mechanisms of spermathecae are limited except for those on honeybees and ants, which show long-term sperm storage by queens. Future studies should compare sperm maintenance systems among solitary and social hymenopteran species with short to long sperm storage durations and conduct phylogenetic analyses to understand the evolution of sperm storage ability in Hymenoptera.

### An anoxic environment improves sperm viability

In this study, we clearly showed that a near-anoxic environment is crucial for sperm viability. The anoxic conditions and consequent sperm immobilization should have significant advantages for sperm physiology in reducing risks from the production of reactive oxygen species and physical cell damage. It is, however, still unknown whether sperm viability is enhanced by sperm immobilization alone or through pleiotropic effects of the anoxic condition. This should become clear when the requirements for sperm immobilization under aerobic conditions are discovered.

Under hypoxic conditions, reactive oxygen species are generated by excess electrons because oxygen is the ultimate electron acceptor in the mitochondrial electron transport system^25^. In ant and honeybee spermathecae, the genes and proteins related to antioxidant function are enriched^26–29^. The antioxidant system may, therefore, protect against oxygen occurring accidentally in the spermathecal reservoir.

Interestingly, sperm cells preserved in an artificial anoxic solution induced by sodium sulfite for more than 4 h differed from those preserved for 2 h. Most of the sperm maintained in anoxic conditions for 1 and 2 h began swimming when aerobic PBS was introduced, but those in anoxia for 4 h did not. This indicates that the physiology of sperm motility was altered within 4 h under anoxia, yet the survival rate was still high. Furthermore, the longer sperm cells were preserved under anoxia, the lower their viability gradually became, where the viability of sperm cells preserved for 0 h and 10 days was > 80% and 27–53%, respectively. This indicates that the near anoxic condition is a key factor for long-term sperm survival, but other factors may also be essential. Therefore, for a complete understanding of long-term sperm storage mechanisms in ant queens, future studies on the role of other factors—such as ions, metabolites, and proteins^29^—are necessary.

## Methods

### Ants

We collected *C*. *osakensis* and *L*. *hayashi* queens from the ground after nuptial flights in Kagawa and Hyogo prefectures in western Japan. The queens were housed in plastic cases with moistened plaster bases at 25 °C. *L*. *hayashi* queens were used only for quantifying oxygen concentration, and *C*. *osakensis* queens at three to ten months after mating were used for sperm motility and viability experiments. We used queens of the same age and from the same collection site in the comparative experiments.

### Measurement of oxygen concentration

We measured oxygen concentrations of the spermathecal fluid and the thoracic and abdominal hemolymph of *C*. *osakensis* queens at one year and *L*. *hayashi* queens at seven months after the beginning of sperm storage (i.e., nuptial flight) using ﻿a calibrated TX3 trace oxygen microoptode (﻿PreSens). The spermatheca was dissected from the queen, and the oxygen probe was inserted into the spermathecal reservoir. The oxygen probe was also inserted into the thorax and abdomen through a small hole created in the intersegment using forceps. To measure oxygen concentrations in *C. osakensis* queens soon after copulation, we dissected queens from 9 to 10 p.m. on the night of the nuptial flight^5^.

### Observation of sperm motility under artificial hypoxic conditions using an O_2_-absorbing agent

To create an artificial hypoxic test solution, an 8-well chamber slide containing 800 µL Dulbecco’s PBS without calcium and magnesium (Nacalai Tesque) and an AnaeroPack® Kenki pouch (Mitsui Gas Co.), which absorbs O_2_ and generates CO_2_, were kept in a sealed plastic bag for 24 h. After 24 h, the oxygen content in the PBS was 0.4–0.5% (n = 3), and the pH had not changed significantly. Sperm cells from the spermathecae of *C*. *osakensis* queens were exposed to the hypoxic PBS. PBS in an 8-well chamber slide placed in atmospheric conditions for 24 h was used as control. A video of the motile sperm was captured using an inverted microscope (Olympus CKX53) and 3CCD digital camera (Olympus DP72). Sperm ejection from the spermathecae and motility observation were performed as quickly as possible (within 2 min) to avoid atmospheric oxygen dissolution in the artificial hypoxic PBS. However, it is likely that sperm cells’ exposure to atmospheric oxygen varied slightly because the time it took to introduce the sperm cells to the solution differed between samples, and oxygen immediately contaminates the PBS on exposure to the atmosphere. Moreover, because the effect of CO_2_ (generated from AnaeroPack Kenki pouch) on sperm immobilization could not be eliminated, we used a test solution containing sodium sulfite as an alternative method to create an artificial anoxic environment.

### Effect of oxygen scavenger, inhibitors of glycolysis and respiration, and pH on sperm motility

We prepared 39.44 mM sodium sulfite (Fujifilm Wako Pure Chemical Corporation), as an oxygen scavenger, in PBS (oxygen concentration of 0%). We also prepared an oxygen-saturated PBS solution containing sodium sulfite solution (oxygen concentration of 21%, matching that of air) by leaving it for a day. The oxygen concentrations in the solutions were verified using a TX3 trace oxygen microoptode. We also prepared 10 µM antimycin (Enzo Life Sciences), oligomycin (Abcam), and FCCP (Cayman chemical) in PBS as inhibitors of the mitochondrial respiratory pathway. In addition, as glycolytic pathway inhibitors, we prepared 50 µM heptelidic acid (Abcam) and 1 mM iodoacetic acid (Fujifilm Wako Pure Chemical Corporation) in PBS. To investigate the effect of pH on sperm motility, 150 mM NaCl with pH 7.1 and 8.6 by adding NaOH and HCl, respectively were also prepared. To prevent change in pH, the NaCl solution was used within 5 min of pH adjustment for each experiment.

A 0.5-mm deep silicone rubber spacer was placed on a slide to keep space between the slide and coverslip. Sperm cells from the spermathecae of *C*. *osakensis* queens were exposed to 20 µL of test solution on the slide and covered with a coverslip, and sperm motility was observed and recorded under a differential interference contrast microscope (Olympus BX53) with a 3CCD digital camera (Olympus DP74).

Sperm motility levels were scored based on the frequency of motile sperm cells as follows: 0, no motile sperm cells; 1, some sperm cells exhibiting motility; 2, intermediate motility, between scores 1 and 3; 3, 80–90% motility; and 4, maximum sperm cells motility. Scoring was performed by a trained observer within 2 min of dissection. In the experiments using an oxygen scavenger and NaCl, the motility scores of all test samples (with one exceptional sample in the PBS control) were either score 0 (immotile) or 4 (motile) (Figs. 2 and 6c). In the experiments using glycolysis and respiration inhibitors, intermediate motility was observed (Fig. 3). To ensure consistent scoring of sperm motility when testing the inhibitors, we performed all observations within 11 days of the experiments.

### Determining pH of spermathecal fluid and sperm

The pH of the spermathecal fluid from *C*. *osakensis* queens was measured using pH paper (pH range 5.5–9.0; Macherey–Nagel). The pH of the hemolymph from a cut on the trochanter of the hind leg was also measured as a control.

To assess the influence of extracellular fluid pH on the sperms’ intracellular pH, the sperm cells from the spermathecae of *C*. *osakensis* queens were stained with BCECF-AM (Dojindo) intracellular pH indicator diluted 1000-fold with pH calibrated buffers, 4.5, 5.5, 6.5, and 7.5 (Intracellular pH Calibration Buffer Kit, Thermo Fisher Scientific), and with the staining buffers containing 10 µM each of valinomycin and nigericin to equilibrate the pH inside and outside of cells, for 10 min. After the sperm samples were centrifuged at 5000 rpm for 5 min and the supernatants removed, a further 20 µL of each buffer was added. From this suspension, a 3 µL aliquot was deposited on a slide, covered with a coverslip, and observed under a fluorescence microscope (Olympus BX53 combined with U-FGW filters). A 3CCD digital camera (Olympus DP74) was used to capture photomicrographs.

### Determining ATP levels in sperm

To determine intracellular ATP, sperm cells from the spermathecae of *C*. *osakensis* queens were introduced to 40 µL of 39.44 mM sodium sulfite in PBS (anoxic condition) and PBS (aerobic condition) for 1 min. Thereafter, the sperm samples were centrifuged at 5000 rpm for 5 min, after which the supernatants were removed, and CellTiter-Glo® Luminescent Cell Viability Assay buffer (Promega) was added. The sperm suspension was then divided into two samples: one for measuring relative luminescence unit (RLU) to determine ATP levels using a CellTiter-Glo Luminescent Cell Viability Assay kit (Promega) and the other for measuring relative fluorescence unit (RFU) to assess DNA content using CellTox™ Green Cytotoxicity Assay kit (Promega). After the samples were prepared according to the manufacturer’s protocol, their luminescence and fluorescence were detected using the GloMax® system (Promega). We confirmed that the values were neither too low to detect nor saturated by checking whether they were within the linear range of the standard curve. To calibrate the ATP content by the number of sperm cells, we calculated values of RLU/RFU.

### Assessing sperm mitochondrial activity

To assess the membrane potential of sperm mitochondria, sperm cells from the spermathecae of *C*. *osakensis* queens were stained using 40 µL of an MT-1 MitoMP Detection Kit (Dojindo), diluted 1000-fold with 39.44 mM sodium sulfite in PBS (anoxic condition), PBS (aerobic condition) and 10 µM FCCP in PBS (negative control) for 15 min. After the sperm samples were centrifuged at 5000 rpm for 5 min and the supernatants were removed, a further 20 µL of each solution was added. Further, 3 µL of the sperm suspension was placed on a slide, covered with a coverslip, and observed under a fluorescence microscope (Olympus BX53 combined with U-FGW filters). Photomicrographs were captured using a 3CCD digital camera (Olympus DP74).

### Assessing sperm viability and motility after preservation under aerobic and anoxic conditions

We exposed sperm cells from the spermathecae of *C*. *osakensis* queens to 40 µL of 39.44 mM sodium sulfite in PBS (anoxic condition) and PBS (aerobic condition) for 6 h, 24 h, 7 days, and 10 days at 23 °C. A 0 h control was also prepared by staining sperm samples immediately after introducing them to PBS. Because 39.44 mM sodium sulfite cannot maintain anoxia in a solution for more than 24 h, sperm samples preserved in anoxic conditions for 24 h, 7 days, and 10 days were kept in a plastic bag with an AnaeroPack Kenki pouch soon after sperm cells were prepared in anoxic PBS created by adding sodium sulfite. Further, sperm cells were stained using a LIVE/DEAD™ Sperm Viability Kit (Thermo Fisher Scientific), which causes live and dead cells to fluoresce green and red, respectively, following the methods of den Boer et al.^30^. Ten microliters of SYBR-14 working solution diluted 50-fold with each solution was added to 10 µL of the sperm suspension and incubated for 10 min. Thereafter, 4 µL propidium iodide was added, and the suspension was incubated for 7 min in dark conditions. Three microliters of the solution was placed on a slide and covered with a coverslip and the observation was repeated four times per sperm sample. Sperm cells preserved for 0 h, 6 h and 24 h were observed using a differential interference contrast microscope to confirm the position of sperm, and a fluorescence microscope (Olympus BX53, combined with U-FBNA, U-FBW, and U-FGW filters) was used to detect the live and dead cells. Photomicrographs were captured using a 3CCD digital camera (Olympus DP74). Fluorescence from sperm cells preserved for 7 days and 10 days was observed and captured using an EVOS™ M7000 Imaging system with GFP and RFP light cubes after phase contrast observation (Thermo Fisher Scientific) to confirm the position of sperm cells. Before observing fluorescence, we selected the observation area using a differential interference contrast microscope or a phase contrast microscope, thus avoiding observer bias in selecting areas of sperm cells showing the expected viability^31^. In total, 150–1000 sperm cells per sample were photographed within 30 min of staining with propidium iodide. Because many sperm cells were degraded in the 7 days and 10 days aerobic condition exposures, no sperm cells were observed in two and three of the six sperm samples, and only 15–477 sperm cells were observed in the remaining sperm samples. The number of living and dead sperm cells was counted on a computer monitor. Because there were few dual-stained sperm cells, they were excluded from the assessment of sperm viability.

We also confirmed whether sperm cells preserved under anoxic PBS conditions using sodium sulfite and AnaeroPack Kenki pouch (for 1, 2, 4, 8, 18, and 24 h) could become motile after exposure to aerobic conditions. A 0.5-mm deep silicone rubber spacer was placed on the slide glass to keep space between the slide and coverslip. Five microliters of the sperm sample in anoxic sodium sulfite solution was deposited on a slide glass and covered with a coverslip. After confirmation of their immobility, 30 µL of aerobic PBS was added, and sperm motility was observed and recorded under a differential interference contrast microscope (Olympus BX53) with a 3CCD digital camera (Olympus DP74).

### Statistical analysis

All statistical analyses were performed using R version 4.1.2^32^. For comparisons between two samples, we used non-parametric Mann–Whitney *U* tests, because for the small sample sizes that we used it was difficult to confirm normal distributions. For multiple comparisons, we used one-way ANOVA with post-hoc Tukey–Kramer tests. To compare sperm motility scores between the control PBS solution and PBS solution containing inhibitors, we used the non-parametric Kruskal–Wallis *H* tests and Steel’s multiple comparison Wilcoxon tests. Differences were considered to be significant at *p* < 0.05.

## Supplementary Information

Supplementary Legends.

Supplementary Movie 1.

Supplementary Movie 2.

Supplementary Movie 3.

## Data Availability

The original data for the graphs in this study are stored on the Figshare repository (10.6084/m9.figshare.22067063.v1).
