# Supplementary material for: Near-anoxia induces immobilization and sustains viability of sperm stored in ant queens
Source: Sci Rep. 2023 Mar 1;13:3029. doi: 10.1038/s41598-023-29705-7 (PMC9977914; doi:10.1038/s41598-023-29705-7)
Supplement: Supplementary file 1 — Supplementary Legends. [file 41598_2023_29705_MOESM1_ESM.pdf]

Supplementary information

Near-anoxia induces immobilization and sustains viability of sperm in ant queens

Ayako Gotoh[1, 2, 3], Mika Takeshima[1], and Ken-ichi Mizutani[4]

[1] Department of Biology, Faculty of Science and Engineering, Konan University, Kobe 658-8501, Japan

[2] Institute for Integrative Neurobiology, Konan University, Kobe 658-8501, Japan

[3] Suntory Rising Stars Encouragement Program in life Sciences（SunRiSE）

[4] Laboratory of Stem Cell Biology, Graduate School of Pharmaceutical Sciences, Kobe Gakuin University, Kobe 650-8586, Japan

Supplementary Movie 1

Sperm motility under hypoxic PBS conditions (created using AnaeroPack® kenki pouch) and aerobic PBS control.

Supplementary Movie 2

Sperm motility in aerobic PBS control and anoxic PBS conditions (created by adding sodium sulfite).

Supplementary Movie 3

Motility of sperm cells preserved in anoxic conditions for 2, 4, and 8 h and subsequently exposed to aerobic PBS solutions.
